# The facilitators and barriers to home-based robotic rehabilitation in India: a pilot feasibility study

**DOI:** 10.3389/fstro.2023.1265702

**Published:** 2024-01-12

**Authors:** Aravind Nehrujee, A. T. Prabhakar, Sathish Balaraman, Rasika Bombatkar, Henry Prakash, Selvaraj Samuelkamaleshkumar, Sanjith Aaron, Suranjan Bhattacharji, S. Sujatha, Sivakumar Balasubramanian

**Affiliations:** ^1^Department of Bioengineering, Christian Medical College Vellore, Vellore, Tamil Nadu, India; ^2^Department of Mechanical Engineering, Indian Institute of Technology Madras, Chennai, Tamil Nadu, India; ^3^Department of Neurological Sciences, Christian Medical College Vellore, Vellore, Tamil Nadu, India; ^4^Department of Physical Medicine and Rehabilitation, Christian Medical College Vellore, Vellore, Tamil Nadu, India; ^5^Department of Physical Medicine and Rehabilitation, Christian Medical College Vellore – Chittoor Campus, Chittoor, Andhra Pradesh, India; ^6^School of Health and Rehabilitation Sciences, The University of Queensland, Brisbane, QLD, Australia

**Keywords:** rehabilitation robot, home-based, usability, serious games, hand, stroke, home trehabilitation, telerehabilitation

## Abstract

**Introduction:**

Robot-assisted rehabilitation has emerged as a promising approach for enhancing motor function in stroke survivors. However, the feasibility and effectiveness of home-based robotic training in this population are underexplored, especially in low/middle-income countries.

**Methods:**

This feasibility study aimed to address this gap by examining the feasibility and effectiveness of independent home-based training using PLUTO, a robotic device for hand training. A total of 7 chronic stroke survivors were recruited, with 5 completing the study.

**Results:**

The results revealed high engagement and adherence to the home-based training program, with participants averaging 1659.8 min of training over 24.8 days. The PLUTO system demonstrated excellent usability and elicited positive user perceptions. Significant improvements were observed in functional outcomes, as evidenced by a noteworthy increase in Fugl-Meyer Assessment scores (mean increase of 6.2 points, exceeding the minimal clinically important difference (MCID) of 5.35 points). Furthermore, participants showed improvements in the ABILHAND measure (mean improvement of 1.24 logits, surpassing the MCID of 0.2 logits) and the Barthel Index (mean increase of 8.8 points).

**Conclusion:**

These findings demonstrate the feasibility and effectiveness of home-based robotic rehabilitation for chronic stroke survivors. This has implications for expanding access to rehabilitation services in low- and middle-income countries, enhancing patient engagement and adherence, and improving functional outcomes. Larger controlled studies are warranted to evaluate the effectiveness of home-based robotic rehabilitation programs.

## Introduction

Stroke profoundly impacts survivors' lives, causing them numerous functional impairments and deficits that impact their quality of life (Wolfe, 2000; Rand and Eng, 2015). A staggering 80% of stroke survivors are estimated to experience some form of upper limb impairment during the early stages of recovery, with around 50% left with chronic impairments (Wade, 1989). These poor post-stroke outcomes are not due to biological constraints but due to lack of administering the appropriate therapy, as demonstrated by recent high-intensity/high-dose studies (Ward et al., 2019; Mawase et al., 2020; Ballester et al., 2022). Thus, conventional post-stroke neurorehabilitation, the current standard of care, underutilises patients' recovery potential.

This underutilization issue is probably more pressing in developing countries like India due to: (a) the large prevalence of stroke survivors requiring neurorehabilitation (Jones et al., 2022), (b) the limited numbers of trained clinicians available in the country (Karan et al., 2019), (c) poor access to high-quality neurorehabilitation care in the tier II/III cities and rural areas (Bright et al., 2018), (d) poor awareness among patients and caregivers about neurorehabilitation (Kamalakannan et al., 2016), and (e) the financial constraints in availing neurorehabilitation services for the majority of patients - as of 2014, only 10% of the population had insurance coverage (Kumar et al., 2012; Ladusingh and Pandey, 2013). These factors limit patients' formal contact with the healthcare system. Particularly in the acute/subacute phase wasting the valuable sensitive recovery period after a stroke (Dromerick et al., 2021).

Decentralized therapy delivered at home or in the community can increase therapy dosage. Thus, unsurprisingly, home-based rehabilitation is integral to conventional neurorehabilitation following a stroke. A paper-based approach for home therapy is a norm where patients/caregivers are trained and given a printed handout detailing a set of exercises/tasks to perform independently at home (Jack et al., 2010; Cheiloudaki and Alexopoulos, 2019; Pishkhani et al., 2020). Contact with a trained clinician during these home therapy programs is only intermittent and prevents patients and caregivers from receiving regular feedback on therapy progress. This often leads to low therapy compliance and high dropout rates, with adherence to these home-based exercises reported to be as low as 28% (Mahmood et al., 2020).

This current landscape underscores the urgent need for innovative solutions for home-based rehabilitation. Rehabilitation robotics technology is a promising option. Robots facilitate intense, high-dose assisted movement training, make therapy more engaging through computer games, can provide regular feedback, track therapy progress, and allow therapy with intermittent therapist supervision. But most existing rehabilitation robots are designed for the hospital or laboratory setting (Turchetti et al., 2014; Qassim and Wan Hasan, 2020), with very few options for home-based therapy due to the robot's size and limited portability. Among these limited options, none have been evaluated in home settings to understand the feasibility of robot-assisted home therapy in India.

### Objectives and hypothesis

The aim of this first-of-its-kind study in India was to evaluate the potential of PLUTO (plug-and-train robot for hand rehabilitation) – a modular, compact, and portable hand rehabilitation robot – as a “therapy extender” by augmenting traditional in-hospital rehabilitation with home therapy. The study's primary objective was to evaluate the feasibility of using PLUTO (Nehrujee et al., 2021) for 4 weeks of independent, at-home hand training amongst chronic stroke survivors. The secondary objective was to evaluate the effect of home-based training with PLUTO on motor impairments and functions in stroke survivors. The hypothesis is that independent home-based hand training for 4 weeks using PLUTO is feasible and useful for chronic stroke survivors in India.

## Methodology

In this pilot study, we utilized a case series design to evaluate the feasibility of PLUTO, a robotic device for home-based hand rehabilitation in chronic stroke patients in India. While we measured clinical outcomes, the primary focus was to understand the practical implementation and acceptance of the technology in the home environment. The robot used in this study, PLUTO, is already integral to regular clinical practice in outpatient settings. The treating clinicians selected the participants in this study, who identified individuals for whom home-based therapy with the robot is likely to be beneficial. These individuals were approached, and their consent was obtained to participate in the study, emphasizing their willingness to engage in home-based rehabilitation using the robot. This approach ensured that the participants represented the population for whom home-based therapy with the robot is a viable option.

### Study participants

Stroke survivors admitted as inpatients or attending outpatient rehabilitation clinics at the Christian Medical College (CMC) Vellore, Tamil Nadu, and CMC Vellore Chittoor campus, Andhra Pradesh, were screened and approached for their willingness to participate in the study. The following inclusion/exclusion criteria were used for recruiting patients into the study:

- *Inclusion criteria:* (i) individuals diagnosed with a first-ever stroke; (ii) must be at least 6 months post-stroke; (iii) should be at least 18 years of age; (iv) should be able to understand simple commands; (v) must have a passive range of motion (PROM) of at least 10 degrees in wrist flexion-extension (WFE), wrist ulnar-radial deviation (WURD), and forearm pronation-supination (FPS), and 4 cm for hand opening-closing (HOC) when assessed using PLUTOI; and (vi) must at least 10 degrees of active range of motion (AROM) in WFE/WURD/FPS, or 4 cm in HOC, when assessed using PLUTO.
- *Exclusion criteria:* (i) individuals with visual and auditory impairments; (ii) individuals with uncontrolled epilepsy; (iii) any fixed contracture and deformity of the hand; and (iv) any psychiatric disorders (v) individuals who could not use the system independently even with the help of a caregiver.

### Intervention

The study intervention involved using a hand rehabilitation robot (PLUTO) to deliver therapy at home for 4 weeks. The details of the device and the training protocol are detailed in the rest of this subsection.

#### PLUTO: plug-and-train robot for hand rehabilitation

**Figure 1:**
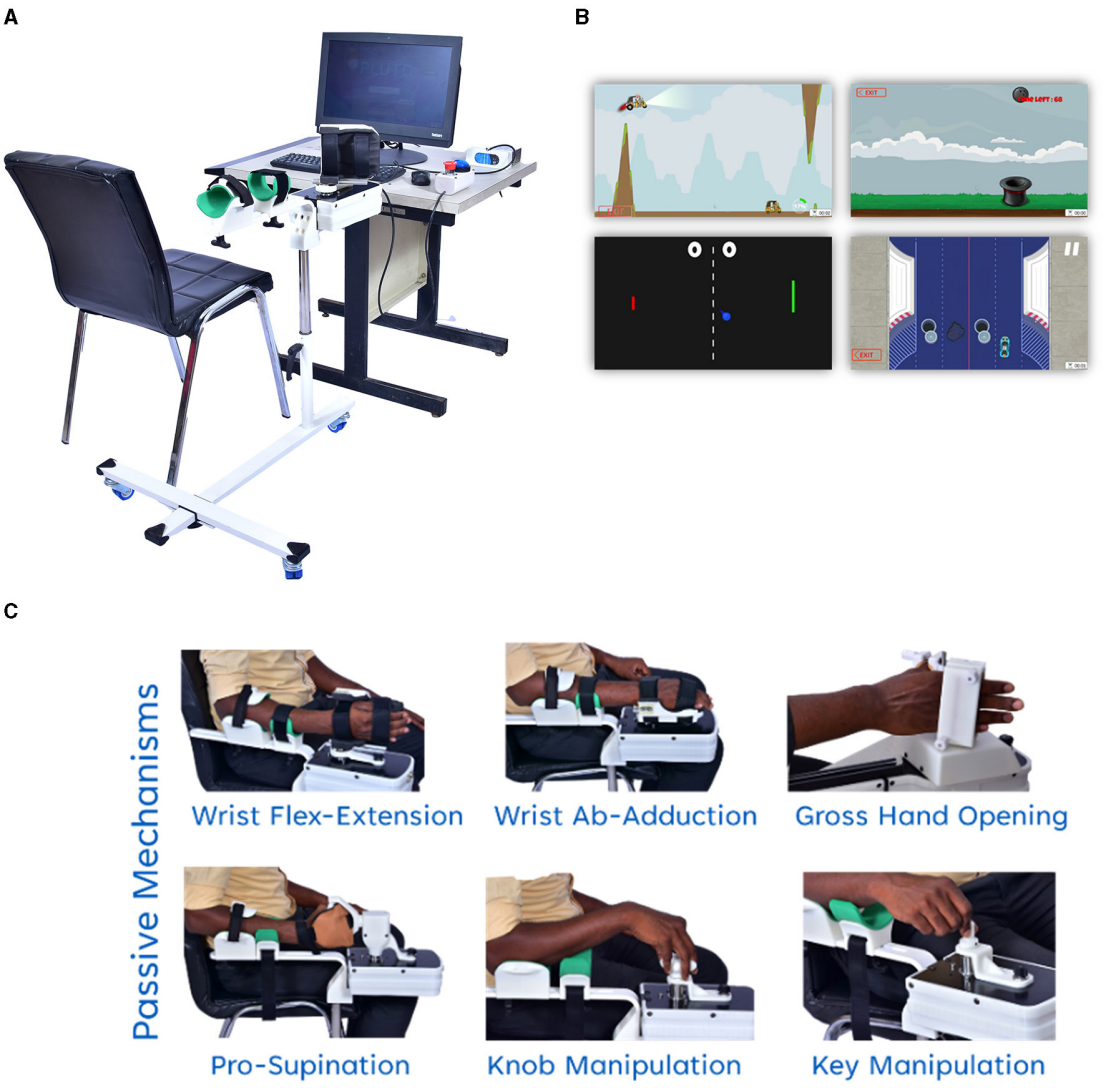
PLUTO device: **(A)** Overall setup of PLUTO **(B)** screenshot of the adaptive computer games **(C)** images of different hand functions Movement trained using PLUTO.

PLUTO is a modular, compact, and portable robotic device designed for assisted hand movement therapy (Nehrujee et al., 2020). PLUTO's motor, electronics, and armrest are mounted on a trolley (Figure 1A). The device is connected to a desktop computer displaying computer games for training different hand functions. The entire setup requires a dedicated table for placing the computer monitor and has an operational footprint of approximately 40-50 square feet (Figure 1A). PLUTO consists of six passive mechanisms targeting different hand movements: wrist flexion-extension, wrist ulnar-radial deviation, forearm supination-pronation, gross hand opening-closing, and two functionally shaped objects – a knob and a key. All training with PLUTO uses adaptive computer games that the user controls through their physical interaction with the robot (Figures 1B, C).

PLUTO allows training in two regimes: active and active-assisted. In the active mode, the device does not assist, allowing participants to perform the movements fully voluntarily. In the active-assisted mode, the robot assists if the patient is unable to complete movement voluntarily. The assistance and the game difficulty are automatically titrated based on the subject's performance.

#### Study protocol

**Figure 2:**
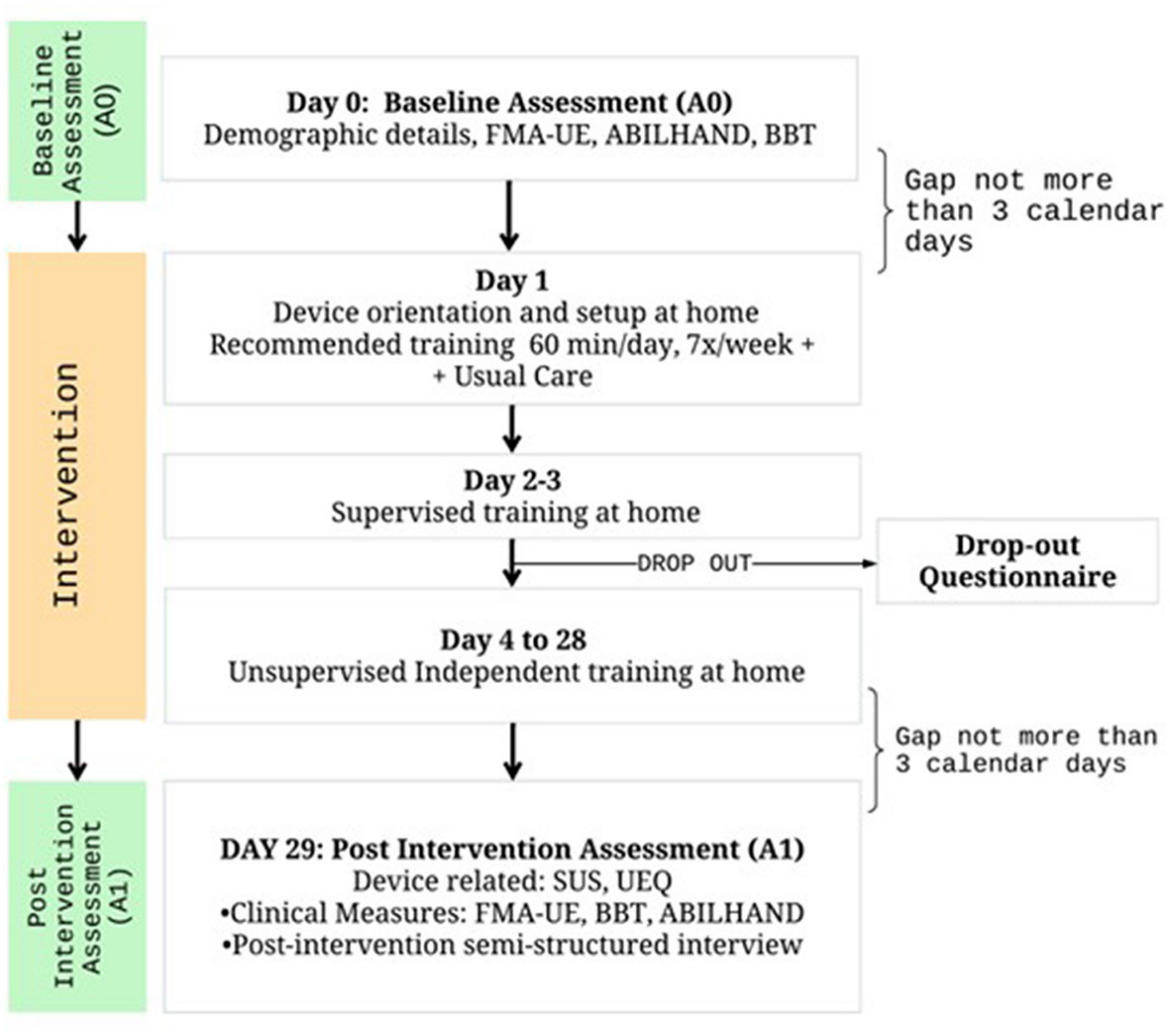
Study flow diagram.

The intervention commenced with an in-clinic demonstration and baseline assessment conducted by an experienced research team, followed by installing PLUTO at the participant's home (Figure 2). A therapist supervised the first 3 days of home therapy to familiarize the patient and the caregiver with the robot and the computer games. The patient and the caregiver were instructed on how to set it up, attach the patient's hand to the robot, and operate the software for choosing games and their difficulty levels. If necessary, supervised training was extended beyond the initial 3 days. After the initial supervised training, patients trained independently with the device, which automatically adjusted the game difficulty and the amount of robotic assistance to challenge the patient sufficiently.

Participants were advised to train with PLUTO for a minimum duration of 1 h per day, 7 days per week for 4 weeks. Caregivers, typically relatives or friends of the patient, played a crucial role in assisting with the daily setup of the device, which included plugging in and out the different mechanisms, attaching the patient's hand to the robot, operating the game software etc.

Participants and caregivers were encouraged to maintain a log of their training durations and were asked to report any adverse event to the research team at the earliest. They were also advised against allowing others to use the device during the study period. Throughout the intervention, participants maintained regular contact with the research team, facilitating the quick resolution of device-related issues and thorough documentation of adverse events such as fatigue or stress.

### Assessment

All subjects underwent evaluations at the onset (A0) and immediately following the home-based intervention (A1). Each participant was subjected to two categories of assessments: (a) Feasibility assessment, encompassing device and protocol-related outcome measures. (b) Clinical assessment, consisting of clinical measures of motor impairment, function, and participation.

The feasibility assessment was conducted exclusively after the intervention period (assessment point A1) via the following outcome measures:

(1) System usability scale (SUS) (Brooke, 1996): This 10-item scale provides a broad perspective of subjective usability experience. Questions are scored on a five-point Likert scale, from “strongly agree” to “strongly disagree.” Responses are converted into a final score ranging from 0 to 100, with a higher score indicating superior usability. Devices garnering a SUS score ≥70 are deemed usable (Bangor et al., 2009).
(2) User experience questionnaire (UEQ) (Laugwitz et al., 2008): Frequently employed as part of a classic usability test to gather quantitative data regarding the participants' system use experience. The UEQ is a 7-point Likert scale comprising 26 questions. These questions are categorized into six sub-scales that evaluate attractiveness, perspicuity, efficiency, dependability, stimulation, and novelty.
(3) Intrinsic motivation inventory (IMI) (Markland and Hardy, 1997): This 12-item multidimensional tool assesses participants' subjective experiences related to the study's target activities. It is scored on a seven-point Likert scale, ranging from “not at all true” to “very true.” A neutral IMI score is four, with a higher score indicating increased motivation. This study employed four IMI subscales: Interest/Enjoyment, Perceived Competence, Tension, and Value/Usefulness.
(4) Technical help: The research team recorded the total number of times they provided technical assistance to the participant or caregiver in using the robot for training from day 4 to day 28. The technical assistance included troubleshooting the software, addressing issues with the plug-in mechanism, resolving hardware/software failures, etc. The team handled most straightforward matters over the phone, while more complex problems necessitated in-person visits to the participant's home.
(5) Device usage statistics: The research team analyzed the usage data collected by the robot during the intervention period, including the number of training days and the daily training duration. It is important to note that the training duration is the active training duration, which was calculated as time spent on different games. Additionally, the device recorded the number of repetitions completed during each session, with one repetition defined as a successful reach to a specific target in different games.

Note that a therapist administered the first three assessments (SUS, UEQ, and IMI) at the end of the study.

The clinical assessment evaluated the impairment and activity domains of the International Classification of Functioning, Disability, and Health (ICF). This study utilized several outcome measures to assess participants' progress and functional outcomes. The Fugl-Meyer Assessment – Upper Extremity (FMA-UE) (Fugl-Meyer et al., 1975) evaluated stroke-related upper extremity impairments. The ABILHAND questionnaire assessed the subjective performance of daily activities (Penta et al., 1998). The Box and Block Test (BBT) measured unilateral gross manual dexterity (Mathiowetz et al., 1985). The Barthel Index (BI) evaluates activities of daily living (Mahoney and Barthel, 1965). These measures provided valuable insights into motor deficits, functional abilities, and independence. MCID values were determined for each measure to identify clinically meaningful improvements: FMA-UE MCID = 5.6 (Hiragami et al., 2019), ABILHAND MCID = 0.26–0.35 logits (Wang et al., 2011), BBT MCID = 5.5 blocks per minute (Chen et al., 2009) and Barthel index MCID = 1.8 (Shah et al., 1989; Hsieh et al., 2007).

We also had a semi-structured interview-based questionnaire to determine if participants noticed any changes in their ability to use their hands for different tasks. The questionnaire evaluated whether performing existing tasks had become easier and whether participants could perform new tasks. Preferences for in-clinic or home-based rehabilitation and general feedback about the program were also recorded.

### Statistical analysis

Due to the exploratory nature of this study and the small number of subjects constituting the case series, conventional inferential statistical tests were deemed inappropriate. Descriptive statistics were employed to summarize the findings from the study. Spearman's correlation analyzed the connection between training dosage (in minutes) and the clinical scales – Fugl-Meyer Assessment (FMA) and ABILHAND – given the small sample size; the statistical significance level was set to be 0.05.

The primary goal of this analysis was to gauge the feasibility of the intervention, which was evaluated using multiple parameters: adherence to the protocol, device usage statistics, need for technical support, subjective usability evaluations, user experience, and motivation. We computed these measures' mean values and ranges, providing a comprehensive picture of the intervention's feasibility.

## Results

This pilot study evaluated the feasibility and effectiveness of independent home-based training using PLUTO on chronic stroke survivors. A total of seven participants were recruited for the study. The intervention involved setting up PLUTO in participants' homes and providing them with training and support to use the device independently.

### Demographic details

The demographic and baseline characteristics of the seven participants are presented in Table 1. Five participants, aged between 18 and 50 years, completed the study. They were all at different stages of their post-stroke journey, ranging from 6 to 42 months. The geographical locations of the participants varied, with three participants residing relatively close to the hospital (6 km), while one participant (P3) was from a rural low-resource setting located more than 100 km from the hospital.

**Table 1:** Participant characteristics.

**Participant**	**Age**	**Gender**	**Time since stroke (months)**	**Side affected**	**Distance from the hospital (km)**
P1	50	F	13	L	< 10
P2	38	M	32	R	< 10
P3	18	M	6	R	>100
P4	44	M	42	R	< 10
P5	34	M	34	R	>10
Median	38		32		6

In addition to the PLUTO intervention, all participants continued their conventional training at home (prescribed exercises approximately 45 min per day, 7 days a week) throughout the study. However, this home-based conventional training was not monitored or logged. Further information about each participant's baseline functional status, including specific hand movements, is provided in the Supplementary Video.

The study had two dropouts. The first occurred shortly after the initial assessment and device installation at the participant's home. This participant developed study-unrelated health conditions that interfered with their ability to use the robot. Subsequently, they pursued an alternative medicine treatment on the advice of another healthcare provider, who had advised against using the robotic device. Consequently, the device was removed from this participant's home after only 3 days of usage. The second dropout happened 2 days into the intervention when this participant, unfortunately, experienced a fall, resulting in a wrist injury. Due to this injury, the participant was required to have their wrist in a cast, thus preventing them from continuing the training with the robot. Given the brief duration of engagement with the intervention, these two participants' data were not included in the analysis.

### Device setup

An engineer and a therapist personally oversaw the device setup to ensure optimal comfort and practicality for each participant. This process took place over the initial 3 days of the intervention period. The tabletop PC and PLUTO were arranged conveniently and comfortably for each participant. The device placement varied based on each participant's living arrangements and preferences to maximize the chances of regular device use. One participant preferred to use the device in the privacy and comfort of their bedroom. Another participant had the device set up in the living room of a relative's house located next door due to infrastructural issues at his home.

###  Device usage statistics

The mean number of days trained by the participants was approximately 24.8 days (out of 28 days), with the total duration of training averaging 1659.8 min (Table 2). This resulted in a mean intensity of 64.53 min of training per day. The mean total repetitions performed by the participants during the training period was ~12750. High intersubject variability was observed in the training duration. Participant P1, for instance, trained the maximum number of days (28 days) and had the highest number of repetitions (24661 reps). On the other hand, participant P5, who trained the least number of days (20 days), had the least number of repetitions (4505 reps). However, P4 demonstrated the highest training intensity, with an average of 99.55 min of training per day.

**Table 2:** Device usage.

	**Days Trained**	**Total duration trained (mins)**	**Intensity (Total duration/days trained)**	**Total repetitions**
P1	28	2360.27	84.30	24661
P2	23	1070.12	46.53	5512
P3	25	1181.55	47.26	8889
P4	28	2787.48	99.55	20192
P5	20	900.00	45.00	4505
Average	24.8	1659.8	64.5	12751.8

**Figure 3:**
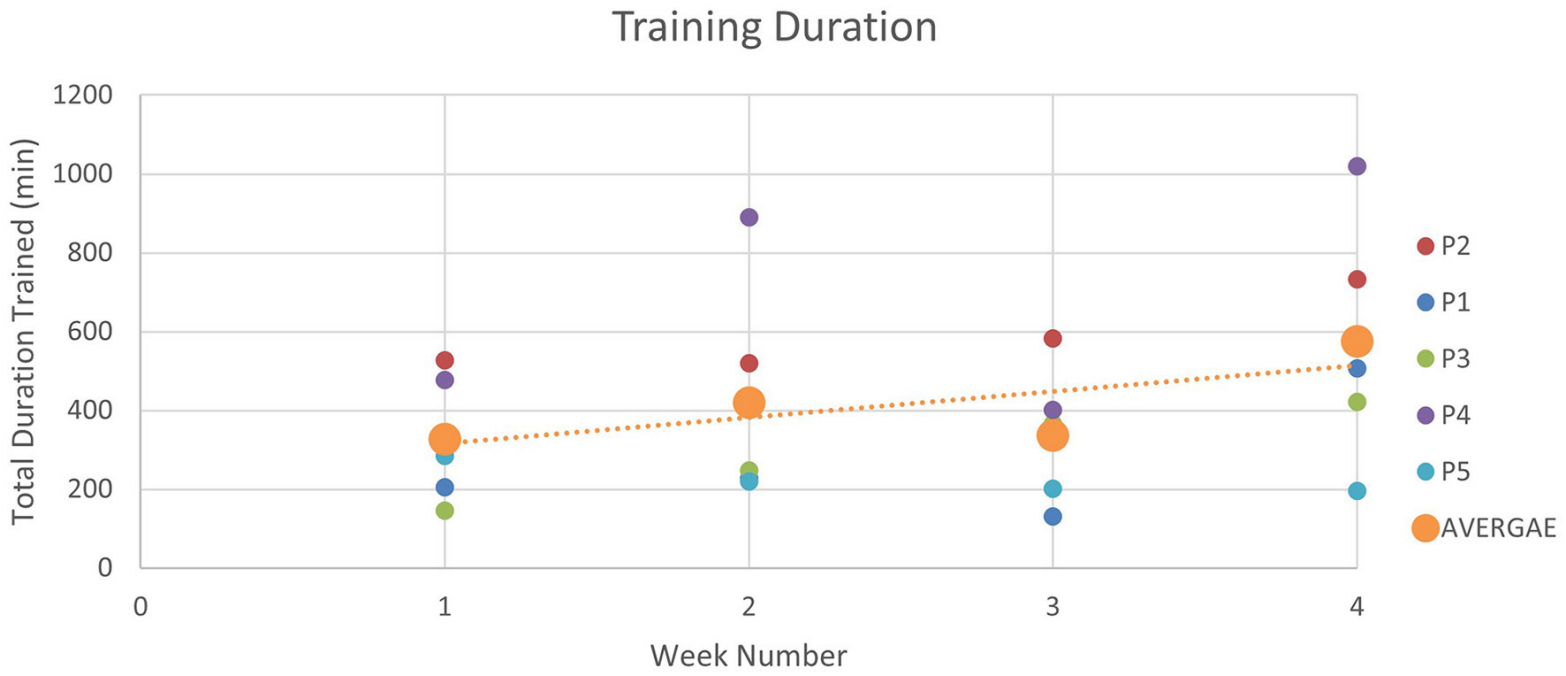
Device usage over 4 weeks: the figure illustrates the duration of robot-assisted training sessions during each week of the intervention. Training durations increased gradually from Week 1 (327.82 min) to Week 2 (420.43 min). In Week 3, the duration slightly decreased to 336.60 min, followed by a significant increase to 575.04 min in Week 4.

Over the course of the study, an increasing trend was observed in the device usage, suggesting that participants were becoming more comfortable with the technology and were increasingly engaged in their home-based rehabilitation (Figure 3).

### Device usability

*System usability scale (SUS):* The mean SUS score across all participants was 81.56, with a standard deviation of 6.78, indicating an “excellent” usability rating according to standard SUS score interpretation. The individual SUS items provide insights into specific aspects of PLUTO's usability (Table 3). For instance, participants rated the statements “I found the robot to be simple” and “I thought the robot was easy to use,” both with a mean score of 3.6 out of 4, indicating that they found the system straightforward and uncomplicated. However, the items “I think that I could use the robot without the support of a technical person” and “I could use the robot without having to learn anything new” received lower mean scores of 2.9, suggesting that participants felt they might need some initial technical support or training to use the system.

**Table 3:** SUS questions-wise results.

**Question**	**Mean**	**Median**	**SD**
I think I would like to use this system frequently	3.1	3	0.54
I found the robot to be simple	3.6	4	0.44
I thought the robot was easy to use	3.6	4	0.44
I think that I could use the robot without the support of a technical person	2.9	3.5	0.83
I found the various functions in this robot were well integrated	2.8	3	0.70
I thought there was a lot of consistency in the robot	3.5	3.5	0.54
I would imagine that most people would learn to use the robot very quickly	3.4	4	0.44
I found the robot very intuitive	3.3	3	0.44
I felt very confident using this robot	3.6	3.5	0.5
I could use the robot without having to learn anything new	2.9	2.5	1
SUS score	81.56	82.5	6.78

*Intrinsic motivation index (IMI):* The IMI comprises of four subscales: Interest/Enjoyment, Perceived Competence, Tension, and Value/Usefulness. The mean score for Interest/Enjoyment was 5.63 (SD = 0.96), indicating a moderate to high level of interest and enjoyment in using PLUTO. For Perceived Competence, the mean score was 6.75 (SD = 0.43), suggesting that participants felt highly competent while using the system. The Tension subscale yielded a mean score of 2 (SD = 1.83), reflecting a low level of tension or stress associated with using the system. Finally, the Value/Usefulness subscale score was 6.88 (SD = 0.22), indicating that participants perceived the system as valuable and useful. These scores indicate a high level of intrinsic motivation when using the PLUTO among participants, with the system perceived as interesting, useful, and not causing undue tension. Participants also felt competent in using the system. Detailed scores are presented in Table 4.

**Table 4:** IMI patient-wise results.

	**Interest/ Enjoyment**	**Perceived competence**	**Tension**	**Value/Usefulness**
P1	6.25	7	5	7
P2	5.25	7	2	6.5
P3	4.25	6	0.5	7
P4	6.75	7	0.5	7
P5	6.75	7	3.5	7
Mean	5.63	6.75	2	6.88
Median	6.25	7	2	7
SD	0.96	0.43	1.8	0.22

**Figure 4:**
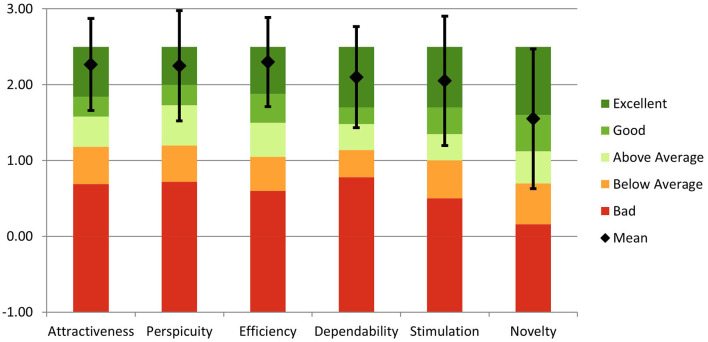
Comparison of Evaluated Product with Benchmark Data. Mean scores and confidence intervals of the User Experience Questionnaire (UEQ) scales are presented, indicating the relative quality of the evaluated product compared to the benchmark (Schrepp et al., 2017). Scores: Attractiveness (2.27), Perspicuity (2.25), efficiency (2.30), Dependability (2.10), stimulation (2.05), and Novelty (1.55).

*User experience questionnaire (UEQ):* The User Experience Questionnaire (UEQ) assessment results for the PLUTO system indicate a positive user perception across multiple dimensions. The system received high scores in attractiveness (2.27), perspicuity (2.25), efficiency (2.30), dependability (2.10), and stimulation (2.05) (Figure 4). These dimensions reflect the system's ability to engage users, provide clear and understandable interactions, offer efficient functionality, ensure reliability, and deliver a stimulating experience. PLUTO performed excellently in these aspects, surpassing the benchmark data (Schrepp et al., 2017) and positioning itself among the top 10% of similar products. However, the scale of novelty received a slightly lower rating (1.55), indicating room for improvement in this area.

### Clinical measures

The clinical outcomes showed changes in some of the scales used in the study (Table 5). The average FMA increased from 22.4 to 28.6 for the study participants, showing 6.2 points increase in FMA; this exceeds the MCID of 5.35; four out of the five participants showed an improvement greater than the MCID in FMA scores. Participants showed an average increase of 5.6 points (out of a maximum of 36), representing a notable 15.5% change in the UPPER EXTREMITY subscale. The WRIST subscale demonstrated an average improvement of 0.6 point (out of a maximum of 10), reflecting a 6% increase from the maximum score, while the HAND subscale showed an average increase of 0.4 points (out of a maximum of 14), corresponding to 2.85% of the maximum score. In contrast, the COORDINATION/SPEED subscale exhibited a minor decline, with participants averaging a decrease of 0.4 points (out of a maximum of 6), equivalent to a −6.67% change. The BBT, however, only had marginal improvement, starting from an average of 0.2 to 1.0 blocks; none of the participants reached the MCID of 4 blocks. The ABILHAND improved by an average of 1.24 logits (−1.33 to −0.08), exceeding the MCID of 0.2 logits, with three participants improving greater than MCID. The Barthel Index also increased on average by 8.8 points (82.8 to 91.6).

**Table 5:** Mean changes in the clinical scales (FMA, Fugl–Meyer assessment; BBT, box and block test; ABIL, ABILHAND ASSESSMENT; BI, Barthel index).

	**FMA**			**BBT**			**ABIL**			**BI**		
	**Pre**	**Post**	**Diff**	**Pre**	**Post**	**Diff**	**Pre (logits)**	**Post (logits)**	**Diff**	**pre**	**post**	**Diff**
P1	20	28	8	0	0	0	−5.918	−2.623	3.295	66	83	17
P2	17	23	6	0	0	0	−1.944	−0.195	1.749	92	96	4
P3	28	33	5	0	2	2	0.192	0.281	0.089	69	87	18
P4	24	33	9	1	3	2	0.471	3.112	2.641	93	100	7
P5	23	26	3	0	0	0	0.570	−0.985	−1.555	94	92	−2
**Mean**	22.40	28.60	6.20	0.20	1.00	0.80	−1.33	−0.08	1.24	82.80	91.60	8.80
**SD**	3.72	3.93	2.14	0.40	1.26	0.98	2.47	1.88	1.77	12.54	6.09	7.68

Improvements were observed among the study participants in the FMA, ABILHAND, and the Barthel Index was more than MCID. The high therapy dosage (~1600min over 28 days) of the current study could be the primary factor behind the positive clinical outcomes in terms of motor impairments and function. Support for the role of dosage can be found by comparing the total number of minutes trained and the clinical improvements in each participant. Participants P1 and P4 had the most improvements in FMA (8 and 9, respectively) and were the ones with the highest therapy dose (2360 min and 2787 min, respectively).

### User feedback

An end-of-study survey was administered to participants after 4 weeks of training (Table 6). When asked if they felt confident training with the system, 3 participants rated it >8/10, one participant 5/10, and one participant 6/10. When asked if they noticed any improvement in the motion of their affected limb, all participants felt there were improvements after robotic training. All subjects felt they could do new tasks at home; subjects reported that tasks became easier after training. Two examples of improvementsmentioned by the subjects: “I'm able to hold the mobile phone”; “I'm able to take a head bath; hold brush confidently.” Finally, three participants preferred using the device at home compared to going to the outpatient clinic for regular therapy: one preferred in-clinic training and one preferred both. All participants felt that therapist feedback was lacking in the current protocol, and it could increase their motivation and compliance. And all participants wanted to keep the device even after the study period.

**Table 6:** Survey responses.

**S.NO**	**QUESTION-Survey for at the end of the home study**	**P1**	**P2**	**P3**	**P4**	**P5**
1	Which therapy did you prefer? A) At-Home Therapy B) In-Clinic	At-Home Therapy	At-Home Therapy	In-Clinic (therapist feedback is important)	Both	At-Home therapy
2	What advantages do you see with home-based therapy?	He can use it whenever he wants- comfortable timing	Timing, Less Travel, Affordable	Comfortable Timing, Convenient	Less travel	Timing convenience
3	What problem do you face in home-based therapy?	Therapist assistance/support is lacking.	Fitting finger in mechanism, polished movement	Technical	Nil	Computer usage
4	Do you think you have become better because of home-based training? (Rate 1−10) 0 – NO; 10 – YES	8	10	8	10	10
	If yes, which activities :		Pronation & Supination, Wrist Flexion & Extension	ADL	Shoulder & elbow joint ROM increased	Trying to write with his impaired hand, holding a bottle
5	How confident are you in using the device [On a scale of 1 (not-confident)- 10 (fully confident)]?	8	6	8	5	10
7	Would you like to keep this device longer? How much longer and why?	Yes, 2 weeks for finger improvement	Yes, 2 weeks	Yes, 4 weeks	Yes, 1 month	Yes
8	How much will you pay monthly for a device like this (INR)?	3000	3000	No	5000	No
9	What is your family income?	~1200k/annum	~1000k/annum	~70k/annum	~360k/annum	~100k/annum

## Discussion

In this feasibility study, 7 participants were recruited, with five completing 4 weeks of independent training at home, and two dropped out of the study due to reasons unrelated to the study. No significant adverse events were reported during the study period. All participants experienced minor technical problems during home use, most of which were addressed over the phone (Table 7). Two instances where a house visit was required and led to a loss of training days were due to (i) a broken emergency switch and (ii) the need for replacement of the hexagonal shaft of the robot's plug-in mechanism.

**Table 7:** Details of help provided: the table provides an overview of the assistance provided to each participant during the intervention.

	**Reason for the assistance**	**Virtual**	**Physical**
P1	Calibration and assessment issue (set neutral old software)−2 times WFE mechanism change Hand rest tightening screw broken Bluetooth connection and Turning ON the robot	4	4
P2	Calibration and assessment issue (set neutral old software) – 2 times FPS mechanism change Assessment and calibration issue Turning ON and Bluetooth connection	4	3
P3	Bluetooth connection Help to use the computer/software after the Windows OS update The emergency switch broken. Assistance given to connect Bluetooth	2	1
P4	Earthing during setting up Bluetooth connection	2	0
P5	Changing the mechanism alone required assistance	4	0

The types of assistance are categorized as physical (red) and virtual over video/audio call (black).

Throughout the course of the trial, all minor issues and bugs encountered with the PLUTO system were successfully resolved. Participants 1 and 2 experienced difficulties with calibration, which were rectified through a software modification that included the addition of a guided GIF on the screen. Bluetooth connectivity and other hardware problems were addressed in real time, significantly reducing the need for physical assistance. Notably, the last participant did not require any physical assistance. These findings underscore the importance of addressing small yet significant practical problems during design testing to enhance the user experience and minimize the need for physical assistance during home-based training.

The device's usability was rated high (~81/100 on SUS). This score is higher than our previous in-clinic feasibility study with PLUTO, where participants rated the SUS at 72.3 (Nehrujee et al., 2021). The excellent usability reported in the current study may be due to (a) the longer time patients spent with the device (4 weeks) compared to 2 sessions in the previous study and (b) the design improvements made to the device since the previous study. The high SUS scores are also further supported by the positive outcomes in the UEQ and the IMI questionnaires. These results provide support for the idea that home-based therapy with technology (like PLUTO) is likely to be well received by chronic stroke patients in India.

The mean usage of the device in this study was ~65 min per day for 4 weeks. This is equivalent or slightly higher than other studies' usage time (Sivan et al., 2014; Bernocchi et al., 2017; Guillén-Climent et al., 2021). This high usage can be attributed to multiple factors, such as ease of use of the device, gamified training, adaptive assistance, round-the-clock easy access to the device, etc.

Although the sample size is small, Spearman's correlation between the number of minutes trained and the change in FMA is 0.9 (*p* < 0.05). The ABILHAND measure also shows a similar trend, but the correlation was lower (0.8, *p* = 0.11) and not statistically significant. Participant P5 had the lowest dosage (900 mins) and showed the smallest gain in FMA, with deteriorating performance on the ABILHAND and the Barthel Index. Although preliminary, these outcomes align with evidence supporting dosage as an important factor in driving recovery (McCabe et al., 2015; Ward et al., 2019).

Interestingly, the significant increase in FMA observed in this study had contributions from the proximal (~16% for the upper extremity) and distal (~6% for wrist and ~2% hand function) subsections of the FMA scale. This generalization of the distal training to the proximal upper limb has been previously reported studies on robot-assisted hand therapy (Hesse et al., 2005; Takahashi et al., 2008; Lambercy et al., 2011). Speculatively, this finding could be attributed to a heightened activation of the sensorimotor cortex, an increased sense of awareness, and improved confidence in using the affected hand (as indicated in Table 6). Such improvements might result from the training of the distal upper limb. Nevertheless, the exact reason for this generalization remains unknown.

Surprisingly, the BBT did not show any change despite a greater than MCID increase in the FMA. One possible explanation could be the modest improvements in the wrist and hand function subscales of the FMA compared to the upper extremity subsection; note that the FMA's upper extremity subsection also assesses pronation-supination, which was trained with PLUTO. The BBT requires proficiency in both proximal and distal control, encompassing proximal shoulder movements and lateral or spherical hand grasp. PLUTO training does not impact these movement components enough to yield significant improvements in BBT scores.

Overall, these outcomes show that technology-aided home-based therapy is a feasible option for decentralizing neurorehabilitation in India for chronic stroke patients. This form of therapy could also lead to significant and useful improvement in upper limb motor impairments and functions. However, its implementation at scale requires understanding the facilitators and barriers to technology-aided home therapy in India. Here, we share some of the factors identified by the current study.

### Facilitators of technology-aided home-based rehabilitation

(1) One of the key facilitators in the current study was the flexibility in the training schedule for the participants. Conducting therapy sessions in the comfort of their homes allowed them to schedule their training according to the conveniences and constraints of their daily life. It also eliminated the need to travel to a rehabilitation center, saving them time, effort, and expenses. This was particularly significant for one participant who resided in a village located >100 km away from the hospital. This demonstrates the potential of technology-assisted home-based protocols to make rehabilitation accessible to individuals in remote areas.
(2) Another important facilitator was that home-based rehabilitation offers the participants a sense of ownership and autonomy. They had control over their training process, actively setting their goals and monitoring their progress with the help of the device. Participants expressed a strong desire to continue using the device even after completing the four-week study, indicating improved engagement and a long-term commitment to their rehabilitation journey.
(3) Prior exposure to technology among patients and caregivers is another important facilitating factor in home-based therapy. This familiarity with different technologies makes them more comfortable to independently use robotic devices and other therapy tools. The user-friendly technology and reliable internet connectivity play a role in alleviating anxiety about using technology for an engaging and useful home-based rehabilitation experience.
(4) The involvement of the caregivers was a crucial facilitator in this study. Caregivers played a supportive role in assisting the participants during their training sessions, ensuring safety, and providing physical assistance when needed. The collaboration between participants and caregivers created a supportive environment that facilitated the successful implementation of the home-based rehabilitation program. The active involvement of caregivers also helped in overcoming technical challenges, as evidenced by the low number of technical visits required throughout the study.

### Barriers to technology-aided home-based rehabilitation

There are several barriers that can hinder the implementation of technology-aided home-based rehabilitation programs. These include:

(1) *Inadequate therapist supervision:* The lack of direct supervision and support from a therapist can impact patients' ability to adhere to therapy plans and track progress. All participants in this study also reported that direct feedback from their training clinician would be valuable. Telerehabilitation sessions with therapists should be incorporated into future plans to address this. Patients also reported a struggle to follow therapy plans independently, making it challenging to reach therapy goals. Progress monitoring and regular performance feedback features must be implemented to address this issue.
(2) *Reliance on caregivers:* Most participants in this study used PLUTO with the assistance of caregivers, making therapy delivery contingent on the availability of the caregiver. Making the device even simpler, easy to don and doff, and an easier user interface to the software would address some of the dependence on the caregiver.
(3) *Technical issues with the device:* Bluetooth connectivity disruptions and limited game variety were among the most reported technical difficulties, which need to be addressed to improve long-term therapy adherence.
(4) *Affordability:* In the post-intervention survey, two participants mentioned that they could not afford home-based therapy if they had to pay for therapy. On the other hand, three participants expressed a willingness to pay a monthly fee of about 3000 INR (the average per capita income in the region was about 18,000 INR per month (tn.gov.in). The lack of finances or insurance to support home-based therapy is an important factor to consider going forward in a developing nation like India. It further emphasizes the need for novel, affordable solutions to ensure equitable access to interventions that can positively impact the quality of life.
(5) *Diseconomies of scale:* Diseconomies of scale present a substantial barrier to the widespread implementation and adoption of home-based robotic rehabilitation programs. These programs require significant initial investments, addressing various logistical hurdles, and the stamina for this to become a sustainable model: a challenge accentuated in low- and middle-income countries (LMIC) such as India. While the current study shows feasibility, its true effectiveness, and implementability remains to be determined. A potential middle ground could be technology-aided community rehabilitation programs, with intermittent tele-supervision from trained clinicians. However, comprehensive economic analyses, tailored to the unique circumstances of LMICs, are essential to ensure the feasibility and cost-effectiveness of scaling up home-based robotic rehabilitation initiatives.

### Limitations of the study

We finally point out the limitations of the current study, a pilot case series. The small sample size limits the generalization of these findings to the population of interest. However, the study's positive outcomes warrant further exploration of this approach in a larger sample size with a more rigorous study design. The clinical assessments were not blinded, which could have played a role in the positive clinical outcomes of the study. However, the observed dose-recovery relationship indicates that the lack of blinding might not fully explain the observed outcomes; the assessing therapist was unaware of the training dosage, which was calculated from the training data only after the post-intervention assessment. The use of robotic assessment of hand function could have provided a more objective measurement of motor impairment and function, which was not carried out in the current study. Another limitation of the restricted nature of training delivered using PLUTO in the current study. PLUTO only focuses on simple single-degree-of-freedom movements of the distal upper limb. This can improve impairments of the trained joints but might not be sufficient to improve ADLs involving more complex movements with multiple degrees of freedom. This could explain the lack of change in the BBT scores. Future studies must explore the addition of a functional training component with other devices to evaluate its effect on motor impairments and function.

## Conclusions

This study demonstrated that individuals with moderate hand impairments post-stroke could safely use PLUTO in a home setting with the help of their caregiver for 4 weeks. Patients could train for about 64 min daily, leading to clinically significant improvements in multiple clinical scales. Most participants preferred home-based robotic training over outpatient training due to its flexibility and reduced travel. This is the first demonstration of the feasibility of robotic home-based training in India and needs to be followed by a larger long-term controlled clinical trial to evaluate the clinical and economic effectiveness of the robotic training at home.

## Data availability statement

The raw data supporting the conclusions of this article will be made available by the authors, without undue reservation.

## Ethics statement

Ethical approval was not required for the studies involving humans because it was conducted as part of a case series and involved an intervention that is already used in regular clinical practice. The studies were conducted in accordance with the local legislation and institutional requirements. The participants provided their written informed consent to participate in this study. Written informed consent was obtained from the individual(s) for the publication of any potentially identifiable images or data included in this article.

## Author contributions

AN: Conceptualization, Formal analysis, Investigation, Methodology, Software, Validation, Visualization, Writing—original draft, Writing—review & editing. AP: Writing—review & editing. SaB: Writing—review & editing. RB: Writing—review & editing, Investigation. HP: Writing—review & editing. SeS: Writing—review & editing. SA: Methodology, Supervision, Writing—review & editing. SB: Supervision, Writing—review & editing, Methodology. SSu: Conceptualization, Funding acquisition, Project administration, Resources, Writing—review & editing, Supervision, Validation. SiB: Conceptualization, Formal analysis, Funding acquisition, Project administration, Resources, Visualization, Writing—original draft, Writing—review & editing.
